# A Qualitative Review of Organizational COVID-19 Communications and Guidance for Pregnant and Postpartum People Who Are Incarcerated

**DOI:** 10.1089/heq.2024.0235

**Published:** 2025-06-05

**Authors:** Ingie Osman, Abaki Beck, Ashley N. Watson, Carolyn Sufrin, Rebecca J. Shlafer

**Affiliations:** ^1^ Department of Pediatrics, University of Minnesota, Minneapolis, Minnesota, USA.; ^2^ School of Public Health, University of Minnesota, Minneapolis, Minnesota, USA.; ^3^ Department of Communication, State University of New York at Geneseo, Geneseo, New York, USA.; ^4^ Department of Gynecology & Obstetrics, Johns Hopkins University School of Medicine, Baltimore, Maryland, USA.

**Keywords:** COVID-19, pandemic, incarceration, pregnancy, postpartum, public health

## Abstract

**Background:**

Each year, thousands of pregnant or postpartum women enter prison and jails across the U.S. During the COVID-19 pandemic, pregnant people who were incarcerated were at increased risk of infection and health complications. Little is known about the role of national public health, medical, and carceral organizations in promoting the health and well-being of pregnant and postpartum people who are incarcerated during the COVID-19 pandemic. The objectives of this study were to assess publicly available COVID-19 communications and guidance from national organizations to better understand guidance for pregnant and postpartum people who were incarcerated during the COVID-19 pandemic.

**Methods:**

This study used documentary qualitative analysis to review publicly available COVID-19 guidance and communications from public health agencies and professional organizations. A total of 27 documents were reviewed, coded, and analyzed across eight organizations.

**Results:**

In the 338 pages reviewed, “pregnancy/postpartum” was coded just 17 times among four organizations. Our review found that mentions of the unique needs of pregnant and postpartum people during the COVID-19 pandemic were mostly absent from organizational guidance.

**Conclusion:**

This analysis calls attention to the gaps in the consideration for pregnant and postpartum people who are incarcerated, particularly in the context of the COVID-19 pandemic. We conclude with a series of recommendations to strengthen the care of pregnant and postpartum people who are incarcerated and promote health equity.

## Background

Pregnancy in prison is a major human rights concern and health equity issue in the United States (U.S.). The number of incarcerated women in the U.S. has increased dramatically over the past several decades; at last count, more than 180,000 women were incarcerated in state and federal prisons and local jails across the U.S.^1,2^ Most of them are of childbearing age, and it is estimated that each year, 3% of women who are admitted to jails and 4% of women who are admitted to prisons are pregnant.^3,4^ Incarceration and maternal health in the U.S. are both marked by systemic racism, resulting in racial disparities in both women’s incarceration rates and in maternal mortality.^1,5^

Carceral systems are responsible for ensuring that incarcerated pregnant people can give birth with safety and dignity. Legally, they are constitutionally required to address pregnant and postpartum individuals’ “serious medical needs.”^6^ Yet, there are no national requirements for pregnancy care in prison and no required systems of oversight for health care services in carceral facilities, which has material impacts on their health. This lack of mandatory standards of care has led to variability in access to, and quantity and quality of, pregnancy care in custody, with numerous reports of substandard pregnancy care.^7^

When it comes to COVID-19 in prison, crowded living conditions, poor ventilation, and unsanitary environments all create conditions in which the virus can easily spread.^8–13^ In particular, the chronic overcrowding of carceral facilities has been shown to be associated with an increase in infectious or communicable diseases.^11^ COVID-19 incidence and mortality rates in prisons and jails have been consistently higher than in the general population.^14,15^ Since March 2020, over 600,000 individuals incarcerated in U.S. correctional and detention facilities have tested positive for COVID-19, although this is likely an undercount.^16^ We have no estimates on the number of COVID-19 cases among pregnant and postpartum people who are incarcerated, highlighting the widespread deprioritization of this population.

People who contract COVID-19 during pregnancy are at increased risk for pregnancy-related health complications and are strongly recommended to take additional steps to reduce their risk of getting COVID-19, such as wearing masks, testing, social distancing, and getting a COVID-19 vaccine.^17–23^ Compared to pregnant people in the general population, pregnant people who are incarcerated have higher rates of trauma exposure, higher rates of substance use, and more mental and physical health issues, impacting both their well-being and the health and well-being of their infants.^17–22^ Prisons are also a driver of reproductive oppression, undermining key tenets of reproductive justice, including the right to have children, the right not to have children, and the right to raise a family with dignity.^23^ These compounding risks of COVID-19 exposure and infection, health conditions, and access to care can influence one’s experience of pregnancy and postpartum during incarceration. Of the limited research to date that has focused on COVID-19 impacts on pregnancy and postpartum care in carceral settings, a survey of 13 prisons and 4 jails found that all facilities suspended prenatal support programs during the pandemic; many facilities reported efforts to continue access to prenatal care (in some cases through telehealth or by increasing off-site appointments); and nearly half of facilities quarantined pregnant people upon their return to the facility from offsite health appointments.^24^ Another study used interviews with pregnant people in one state prison to describe how the pandemic and prison practice/policy changes worsened already intolerable physical and psychosocial conditions for pregnant people.^25^

Throughout the pandemic, national organizations, such as the Centers for Disease Control and Prevention (CDC), the American College of Obstetricians and Gynecologists (ACOG), the American Medical Association (AMA), and more, released guidance, recommendations, and information specific to pregnant people in the general population, aiming to increase protection and promote well-being.^23,26–28^ Simultaneously, the CDC and other national health organizations have put out specific guidance and calls to action for carceral facilities to minimize the harms of COVID-19 among incarcerated people and staff.^13,29,30^ However, little is known about the role of national public health, medical, and carceral organizations in promoting the health and well-being of pregnant and postpartum people who are incarcerated during the COVID-19 pandemic.

Given this critical intersection of incarceration and pregnancy, it is essential to have more robust knowledge about guidance for this population during public health emergencies, such as the COVID-19 pandemic and beyond, to promote health equity. Through this study, we assess publicly available COVID-19 communications and guidance from eight national health and correctional organizations to better understand whether the provision of guidance for people who are incarcerated included specific guidance for pregnant and postpartum individuals and key themes that emerged from the guidance.

## Methods

We conducted an in-depth qualitative analysis of publicly available COVID-19 guidance and communications from public health agencies and professional organizations (e.g., public statements, publicly available guidance). Using documentary analysis methods similar to Dallaire et al., Gatta et al., and Celinska & Cheboubi, members of the research team reviewed the websites of eight public health, medical, and correctional organizations for COVID-19-related information specific to correctional facilities published anytime between March 2020 and March 2024 (see Table 1).^31–33^ The focus of this analysis was on national organizations providing COVID-19-related guidance relevant to this population, so state-specific organizations were not included in this review. Organizations were selected based on authors’ knowledge of organizations that issue health-related guidance on the care and treatment of incarcerated populations. Authors reached consensus and selected these eight organizations that were found to be the most relevant to this population; authors selected four medical/public health organizations and four corrections organizations to review for this study. In their search, research team members used search terms such as “COVID-19,” “prisons,” “jails,” “pregnant,” “postpartum,” “incarceration,” “coronavirus,” “COVID,” and “pandemic” and manually reviewed websites to systematically capture relevant information for analysis. Documents that were reviewed also included any archived guidance or materials that were still accessible through website searches (e.g., archived CDC guidance for correctional facilities); however, they did not include books or other long-form publications that were not available on webpages.

**Table 1.** Summary of Themes, by Organization

Org. Type	Org. Title	No. of documents reviewed	No. of pages of documents reviewed	No. of mentions of pregnancy in prison	Summary of themes
Government	Centers for Disease Control and Prevention (CDC)	8	96	1	- Nothing specific to pregnant people in prison - One press release noted people with higher risk have special considerations and mentioned pregnancy as a high-risk category, but did not explicitly mention incarcerated pregnant people
Corrections	American Correctional Association (ACA)	1	3	0	- Nothing specific to pregnant people in prison
	American Jail Association (AJA)	4	28	0	- Nothing specific to pregnant people in prison
	National Sheriff’s Association (NSA)	3	16	0	- Nothing specific to pregnant people in prison
	National Commission on Correctional Health Care (NCCHC)	4	137	4	- Pregnancy mentioned as a “high risk” category for COVID-19
Medical/Public Health	American Medical Association (AMA)	4	13	0	- Nothing specific to pregnant people in prison
	American Public Health Association (APHA)	1	13	2	- Nothing specific to pregnant people in prison, but mentioned impact of parental incarceration on children
	American College of Obstetricians and Gynecologists (ACOG)	2	25	10	- Comprehensive perinatal care, including breastfeeding support, should be guaranteed and available without barriers like co-pays - Vaccinations should be available but not mandatory - Solitary confinement should not be used in lieu of medical isolation - Rights and autonomy of incarcerated pregnant and postpartum people should be maintained - Decarceration proposed as main solution

After identifying recommendations and guidance, three reviewers took screenshots of all webpages or, when available, downloaded PDFs, which were then uploaded to Dedoose for coding and analysis. Twenty-seven documents/webpages across the eight organizations were identified and coded (see Appendix A1 for a list of documents and Table A1 for a codebook). The documents totaled 338 pages in length. Members of the research team then used *a priori* codes that were developed based on an initial review of the documents, focusing on domains such as health services and social support (Table A1). Each document was assigned two coders. All coders met on a weekly basis to reflect on key learnings and takeaways and talk through any questions that arose during the coding process. In separate meetings, each pair of coders met to reconcile any conflicts in their assigned transcripts and reach consensus; inter-coder reliability was not calculated for this study. Once the coding process was completed, the team met to identify common themes, patterns, and relationships among the codes.

**Table A1.** Codebook

Code	Definition
Population-specific codes	
Trauma-informed care	This code describes the mention of trauma-informed care as it relates to the COVID-19 pandemic and the carceral system. Trauma-informed care is a strengths-based framework that is grounded in an understanding of and responsiveness to the impact of trauma.
Pregnancy/postpartum	This code describes the mention of pregnancy or postpartum in COVID-19 guidance, specifically focused on incarcerated populations. It may encompass certain considerations that may be taken into account for pregnant and postpartum who are incarcerated.
Parenting	This code describes the mention of parenting in COVID-19 guidance, specifically focused on incarcerated populations. It may encompass certain considerations that may be taken into account for parents of minor children who are incarcerated.
Justification for practice/policy changes	
Support of mental/emotional wellbeing	This code describes the goal of supporting incarcerated individuals’ mental and emotional wellbeing as justification for COVID-19-related practice or policy changes.
Curbing the spread of infectious disease	This code describes the goal of curbing the spread of COVID-19 and other infectious diseases as justification for COVID-19-related practice or policy changes.
Ease of facility operations/staffing	This code describes the goal of aiming to support facility operations and staff as justification for COVID-19-related practice or policy changes.
Health Services	
Access to health services	This code describes guidance that works to ensure people continue to have access to health services during policy and operational changes resulting from the COVID-19 pandemic.
Social Support	
In-person visitation	This code describes guidance that works to ensure people continue to have access to in-person visitation during policy and operational changes resulting from the COVID-19 pandemic.
Communication	This code describes guidance that works to ensure people continue to have access to communication (e.g., phone calls, video visits) during policy and operational changes resulting from the COVID-19 pandemic.
Access to programming	This code describes guidance that works to ensure people continue to have access to programming during policy and operational changes resulting from the COVID-19 pandemic.
COVID-19 Practice/Policy Recommendations	
COVID-19 quarantine and isolation	This code describes any COVID-19 quarantine and confinement practices (e.g., isolation upon intake, medical isolation, unit lockdown, etc.) implemented by carceral facilities or medical facilities that impacted pregnant and/or postpartum individuals.
COVID-19 release practices	This code describes release practices (e.g., Conditional Medical Release) for pregnant and/or postpartum individuals that were implemented as a result of the COVID-19 pandemic.
Other preventative measures	This code describes any recommendations for practice/policy changes around COVID-19 (e.g., testing, masking, handwashing, etc.) that may have impacted facility operations and/or pregnant and/or postpartum individuals.
COVID-19 vaccination	This code describes any mention of COVID-19 vaccination for pregnant and/or postpartum individuals, facility staff, or volunteers.
Other	
Other	This code encompasses anything noteworthy that we want to capture, that does not fall into any of the above codes.

## Results

In the 338 pages across 27 documents of the eight organizations, “pregnancy/postpartum” was coded just 17 times by four organizations. A passage was coded if it explicitly named pregnant or postpartum incarcerated people, whether or not there was an associated recommendation. Ten of those codes were by ACOG, and the other mentions—by the CDC, National Commission on Correctional Health Care (NCCHC), and American Public Health Association (APHA)—were cursory: most commonly, the focus of these mentions was listing pregnancy as one example of individuals who are at “high risk” of contracting severe COVID-19, rather than to provide specific recommendations for pregnant or postpartum people in correctional settings. For example, one mention of pregnancy on an NCCHC document simply said “Pregnancy?” at the end of a bullet point list of those who are high risk, while another NCCHC document said, “Currently, no data suggests pregnant women are at higher risk for contracting COVID-19.” Seven documents—three from the National Sheriff’s Association (NSA) and four from NCCHC—had no mention of pregnancy and were thus not coded for “pregnancy/postpartum.” See Table 1 for a summary of findings.

ACOG was the only organization that provided specific recommendations on pregnancy in prison during the pandemic. Unlike other organizations, they created a document specifically for that purpose, titled “COVID-19 Public Health Considerations for Pregnant and Postpartum People Who Are Incarcerated,” published in 2021 and revised in 2023. These recommendations recognized that various intersecting factors—incarceration status, pregnancy status, and institutional racism—mean special considerations need to be taken to care for pregnant incarcerated people, who are at increased risk of contracting severe COVID-19 infection. Broadly, the guidance focused on access to health care while incarcerated and the treatment and rights of pregnant incarcerated people. Their first and primary recommendation was that pregnant people should not be incarcerated “whenever possible and consistent with public safety.” They also provided longer-term recommendations, namely, decarceration, elimination of pretrial detention, and allocation of resources for alternatives to incarceration.

Their remaining recommendations fell into three categories. First, ACOG discussed how policy and practice changes created systemic strains impacting prenatal and postnatal care. Even if a pregnant incarcerated person did not contract COVID, they still could be impacted by policy and practice changes during the pandemic. To address this, they recommended carceral facilities actively work to ensure access to comprehensive perinatal care, including breastfeeding support, and that it be available without barriers (e.g., no copays and telehealth if in-person visits were restricted due to COVID protocols). However, there were no explicit recommendations focused on labor, delivery, and postpartum specifically. Second, they strongly urged that solitary confinement not be used for medical isolation, especially for pregnant and postpartum incarcerated people. They noted that pregnant people may be deterred from seeking medical attention if they know it means they will have to quarantine upon returning to the prison from an external health care facility. Finally, ACOG made several recommendations around COVID-19 vaccinations. Namely, they assert that though vaccines should be made available to pregnant and postpartum incarcerated people, individual autonomy and choice to be vaccinated or not should be prioritized.

With the exception of ACOG’s nuanced discussion of pregnancy and postpartum inside carceral facilities, mentions of the unique needs of pregnant and postpartum people were mostly absent from the CDC and other organizations’ guidance. Although pregnancy-specific guidance/recommendations were not mentioned other than by ACOG, there were still important themes that emerged about incarcerated populations more broadly. Many organizations, particularly correctional organizations such as the American Correctional Association (ACA) and American Jail Association (AJA), did not create their own guidance, instead linking to CDC guidance. ACOG also referenced CDC recommendations for COVID-19 prevention. Thus, it is important to understand what the CDC recommended and when, as many carceral facilities were turning to them for guidance.

First, there was a focus on Department of Corrections staff and prison and jail operations as opposed to the rights and health of incarcerated people. For example, the CDC’s 2020 guidance focused on preparation for corrections staff and operational challenges, like managing increased staff sick days, and the importance of communicating pandemic policy changes with staff. Second, the CDC’s language on quarantine and isolation shifted depending on the time during the pandemic. For example, in the CDC’s 2020 guidance for correctional facilities, they discussed how to medically isolate individuals. In 2023 guidance, the CDC highlighted that quarantining was no longer recommended for the general public and may cause negative disruptions to in-person visiting and access to programming for incarcerated people. Third, CDC guidance discussed how the pandemic might impact visitation and programming. CDC guidance also explicitly mentioned how the pandemic and changes in policy and operations may impact the mental health of incarcerated people and staff. For example, the 2023 guidance, unlike the 2020 guidance, said to prioritize access to in-person visiting even if COVID transmission was high and that virtual visiting should not be considered an alternative. Through this analysis, it was evident that guidance shifted over time as new information related to the pandemic and its impacts emerged. A final theme that was not in CDC guidance but is still notable is that some organizations—including APHA and ACOG—discussed longer-term solutions like decarceration and abolition. APHA explicitly mentioned “abolition of carceral systems,” advocating heavily for community-based alternatives to incarceration and decarceration, and had less emphasis on what carceral systems could do to support currently incarcerated people.

## Discussion and Recommendations

This document analysis calls attention to the clear gaps in the consideration and care for pregnant and postpartum people who are incarcerated, particularly in the context of the COVID-19 pandemic. It is evident from this review that the federal agency and the majority of organizations included in this analysis did not focus on providing timely COVID-19-related guidance for this specific population, ignoring the unique needs of pregnant and postpartum people behind bars and the distinct susceptibility of pregnant individuals to COVID-19. This omission highlights a clear gap and missed opportunity to promote health equity among those disproportionately impacted by the pandemic. Given these findings, we conclude with a series of recommendations to strengthen the care of pregnant and postpartum people who are incarcerated in the context of the COVID-19 pandemic and other future public health emergencies that may arise.

1) Create tailored guidance for this population, keeping in mind the unique experiences of pregnant and postpartum people behind bars. A starting point for recommendations around access to medical care, isolation, vaccination, and more can be found in ACOG’s “COVID-19 Public Health Considerations for Pregnant and Postpartum People Who Are Incarcerated.”^34^ Consult a wide range of professionals, as well as formerly incarcerated people who have experienced pregnancy and postpartum while inside, in crafting guidance and recommendations.^35^ Involving a range of stakeholders from different disciplines can strengthen recommendations and work to minimize any unintended consequences while ultimately working to protect the health and well-being of this population.^36,37^
2) Employ both a top-down and a bottom-up approach when developing guidance for this population. In our analysis, we saw that the majority of organizations we reviewed cited the CDC for their guidance and recommendations. If the CDC is not calling out this population in their guidance, many state and local facilities may follow suit. We recommend that federal public health and other national medical and correctional agencies create and highlight specific guidance for pregnant and postpartum people who are incarcerated; in doing so, we anticipate that many local correctional facilities will follow suit in adopting this guidance.

While a top-down approach and leadership in this space are important, we recommend creating channels that support a bottom-up approach, as well. With a bottom-up approach, we encourage local prisons and jails to bring up practice-based issues they have encountered in their work and collaborate with state agencies to share and problem-solve; this is particularly important when considering that each carceral facility has different capacities and needs that may differ from generalized guidance. We also encourage collaboration with directly impacted individuals, centering their expertise and wisdom. Creating these channels for timely communication of on-the-ground, day-to-day issues, as well as uplifting the voices of those most impacted, will allow state and federal agencies to hear what is happening and allow for feedback loops to be created that support policy development and implementation.

3) Provide technical assistance in disseminating and implementing guidance in a timely manner. Amidst rapidly evolving public health emergencies, such as the COVID-19 pandemic, federal agencies need to consider how to get these guidelines to different states, counties, and facilities in a timely and effective way. While it is important to create guidance for pregnant and postpartum people who are incarcerated, the guidance needs to make its way into facilities to be impactful. We recommend that health departments host regular calls with carceral facilities to provide up-to-date information, practical resources and support, and provide space for feedback loops to occur. Proactively creating these spaces for rapid dissemination and communication is essential so agencies and organizations are prepared for situations that require rapid response. Within carceral settings, in particular, it is challenging to provide oversight when these systems are closed-off from others in the community.^38^ We encourage local and state public health departments, with support from federal public health and medical agencies, to provide oversight to support the adoption of guidance and recommendations for this population.
4) Work to release pregnant and postpartum people from carceral facilities. Ultimately, evidence shows that carceral settings are not conducive to health and well-being.^39^ Some of the organizations we reviewed, such as APHA and ACOG, recommend decarceration as a strategy to improve the health and well-being of individuals.^34,40^ In line with these organizations and toward health equity, we recommend that whenever possible, states should employ tactics that work toward decarceration, specifically prioritizing the diversion or release of pregnant and postpartum people who are incarcerated. States that employed decarceration strategies during the pandemic demonstrated that it is possible to employ decarceration as a strategy for health promotion moving forward. Recognizing the detrimental health consequences of incarceration, we encourage national organizations, such as the CDC, to adopt decarceration as a public health strategy and recommend it as a tool towards achieving health equity.^40–42^ When decarceration opportunities are pursued, we recommend that they be accompanied by comprehensive, wrap-around re-entry supports (e.g., access to housing, transportation, and health care) to help minimize the many barrier individuals face upon re-entry.

## Strengths and Limitations

This study has multiple strengths—no analysis, to our knowledge, has examined and documented national guidance specifically for pregnant and postpartum people who were incarcerated during the pandemic. This study also incorporates perspectives from multiple disciplines, including public health, medicine, and corrections, allowing for a multifaceted look at guidance for incarcerated populations during this time. However, several limitations of this study should be considered. First, our analysis was limited to several national organizations but did not include searches of the Federal Bureau of Prisons, individual DOC or jail facilities, or state/local health department websites. It is possible that some smaller agencies and institutions created their own policies for pregnant and postpartum people who were incarcerated in response to the COVID-19 pandemic. Additionally, our study did not analyze any long-form content that organizations and agencies have published (e.g., books/manuals) that were not available on webpages. As such, there may be underestimates in the number of agencies and organizations across the country that focused on pregnant and postpartum people who are incarcerated in their guidance.

## Health Equity Implications and Conclusion

This document analysis highlights the absence of guidance from national public health, medical, and carceral organizations for pregnant and postpartum people in prison during the COVID-19 pandemic and provides recommendations to better support the health and well-being of this population. This is a symptom of the broader neglect of pregnant individuals in the carceral system and of incarcerated individuals in national maternal health conversations. Understanding how and when organizations recognize and provide support for pregnant and postpartum people who are incarcerated is critically important in creating practice and policy that is responsive to the impacts of the COVID-19 pandemic and that promotes health equity.

The COVID-19 pandemic, its impact in carceral settings, and the lack of attention to this population highlighted by this analysis illustrate the inequitable care and treatment for pregnant and postpartum people in prison more broadly. When preparing for public health emergencies, planning health promotion activities, or more generally providing care to this population, it is imperative that there are clear and timely channels for dissemination, implementation, and oversight when guidance is put into place. There are many lessons to be learned from the COVID-19 pandemic, especially within carceral settings, and these lessons need to be adopted by national public health, medical, and carceral organizations, and more broadly, in preparation for future COVID-19 surges, emerging infectious diseases, other disaster preparedness and planning, and broader efforts to support the health and well-being of this population.

## Appendix

### Appendix A1. List of documents reviewed.

American College of Obstetricians and Gynecologists. (2023, July 25). *COVID-19 public health considerations for pregnant and postpartum people who are incarcerated.* https://www.acog.org/clinical-information/policy-and-position-statements/position-statements/2021/covid-19-public-health-considerations-for-pregnant-and-postpartum-people-who-are-incarcerated
American College of Obstetricians and Gynecologists. (2023, October 4). *COVID-19 FAQs for obstetrician-gynecologists, obstetrics*. https://www.acog.org/clinical-information/physician-faqs/covid-19-faqs-for-ob-gyns-obstetrics
American Correctional Association. (n.d.). *Coronavirus COVID-19 resources and information*. https://www.aca.org/ACA_Member/ACA/ACA_Member/Healthcare_Professional_Interest_Section/Coronavirus_COVID.aspx
American Medical Association (2020, May 15) The impact of COVID-19 on the nation’s incarceration system. https://www.ama-assn.org/delivering-care/population-care/impact-covid-19-nations-incarceration-system
American Medical Association (2020, May 20). What’s driving COVID-19 in prisons and jails–and how to fix it. https://www.ama-assn.org/delivering-care/public-health/what-s-driving-covid-19-prisons-and-jails-and-how-fix-it
American Medical Association (2020, June 18). Ethics Talk Videocast Transcript–Caring for Incarcerated Individuals During COVID-19 Pandemic. https://edhub.ama-assn.org/ama-journal-of-ethics/video-player/18518254
American Medical Association (2020, November 17). *AMA policy calls for more COVID-19 prevention for congregate settings.* https://www.ama-assn.org/press-center/press-releases/ama-policy-calls-more-covid-19-prevention-congregate-settings
American Jail Association. (n.d.). *COVID-19 Resources* webpage https://www.americanjail.org/covid-19%20resource
American Jail Association. (n.d.). *Operations and workplace strategies* webpage https://www.americanjail.org/operations workplace strategies
American Jail Association. (n.d.). *Resource guide and considerations for reopening a detention facility under COVID-19 restrictions*. https://www.americanjail.org/files/Resource%20Guide%20and%20Considerations%20for%20Reopening%20Under%20COVID-19.pdf
American Jail Association. (2021, August 24). *New Correctional Indication for Monoclonal Antibody (Regen-COV) for SARS-CoV2.* https://www.americanjail.org/files/Regen-COV%20Information%20Sheet%208_24_21%20release%20*(004).pdf*
American Public Health Association. (2020, October 24). *Advancing public health interventions to address the harms of the carceral system.* https://www.apha.org/policies-and-advocacy/public-health-policy-statements/policy-database/2021/01/14/advancing-public-health-interventions-to-address-the-harms-of-the-carceral-system
Centers for Disease Control and Prevention (2020, March 23). *Interim Guidance on Management of Coronavirus Disease 2019 (COVID-19) in Correctional and Detention Facilities.* https://www.ncchc.org/wp-content/uploads/CDC_Correctional_Facility_Guidance_032720.pdf
Centers for Disease Control and Prevention (2023, May 11). Guidance on Management of COVID-19 in Homeless Service Sites and in Correctional and Detention Facilities. https://archive.cdc.gov/www_cdc_gov/coronavirus/2019-ncov/community/homeless-correctional-settings.html
Centers for Disease Control and Prevention. (2024, March 1) CDC updates and simplifies respiratory virus recommendations. https://www.cdc.gov/media/releases/2024/p0301-respiratory-virus.html
Centers for Disease Control and Prevention. (2024, March 1) *Respiratory Viruses Guidance* webpage. https://www.cdc.gov/respiratory-viruses/guidance/index.html
Centers for Disease Control and Prevention. (2024, March 1) *Respiratory Viruses and Young Children* webpage. https://www.cdc.gov/respiratory-viruses/risk-factors/young-children.html
Centers for Disease Control and Prevention. (2024, March 1) *Respiratory Viruses and Pregnancy* webpage. https://www.cdc.gov/respiratory-viruses/risk-factors/pregnant-people.html
Centers for Disease Control and Prevention, Office of Readiness and Response. (2024, March 7). *CDC Respiratory Virus Guidance: What it Means for Correctional and Detention Facilities.* Webinar presentation slides. https://bja.ojp.gov/doc/cdc-virus-guidance-presentation.pdf
Centers for Disease Control and Prevention, Office of Readiness and Response. (2024, March 7). *CDC Respiratory Virus Guidance: What it Means for Correctional and Detention Facilities.* Webinar transcript. https://bja.ojp.gov/doc/cdc-virus-guidance-transcript.pdf
National Commission on Correctional Health Care and Louisiana Department of Corrections. *Women and Addiction: Gender Specific Care Models Care Models.* Webinar presentation slides. https://www.ncchc.org/wp-content/uploads/320-Edgerton-Women-Addiction.pdf
National Commission on Correctional Health Care and Major County Sheriffs of America. *COVID-19: Steps to an Effective Response.* Webinar presentation slides. https://www.ncchc.org/wp-content/uploads/COVID-19-NCCHC-Slides.pdf
National Commission on Correctional Health Care and Residential Substance Abuse Treatment (RSAT) Training and Technical Assistance. *Preparing People for Reentry from Jails and Prisons During the COVID-19 Epidemic.* Webinar presentation slides. https://www.ncchc.org/wp-content/uploads/RSAT_C-19_Release_dbak.pdf
National Commission on Correctional Health Care and American Academy of Pediatrics. *COVID-19 in Juvenile Justice Settings: A Roundtable Discussion.* Webinar presentation slides. https://www.ncchc.org/wp-content/uploads/JJS-covid-roundtable-060520.pdf
National Sheriffs’ Association. (2020, April 14) *Industry Action Group* webpage. https://www.sheriffs.org/coronavirus/iag
National Sheriffs’ Association. (n.d.) *NSA Mask Project* webpage. https://www.sheriffs.org/coronavirus/masks
National Sheriffs’ Association. (n.d.) *Federal Bureau of Prisons COVID-19 Action Plan.* https://www.sheriffs.org/coronavirus/bop

## Abbreviations Used

ACA: American Correctional Association
ACOG: American College of Obstetricians and Gynecologists
AJA: American Jail Association
AMA: American Medical Association
APHA: American Public Health Association
CDC: Centers for Disease Control and Prevention
COVID-19: Coronavirus Disease 2019
DOC: Department of Corrections
NCCHC: National Commission on Correctional Health Care
NSA: National Sheriff’s Association
U.S.: United States
